# Editorial: Genetic dissection and improvement of crop quality and stress adaptation

**DOI:** 10.3389/fgene.2025.1694599

**Published:** 2025-09-30

**Authors:** Liwei Zheng, Hangkai Zhang, Songwen Zhang, Yingpeng Hua, Lihu Wang

**Affiliations:** ^1^ School of Agricultural Sciences, Zhengzhou University, Zhengzhou, China; ^2^ Department of Pharmacology, University of Washington, Seattle, WA, United States; ^3^ School of Landscape and Ecological Engineering, Hebei University of Engineering, Handan, China

**Keywords:** agriculture, crop quality, stress adaptation, genetic dissection, molecular breeding

## Introduction

Global food systems are at a critical juncture. Rising temperatures, erratic rainfall, soil salinity, and emerging pathogens are eroding crop productivity at unprecedented rates. At the same time, a growing population—expected to reach 9.7 billion by 2050—demands more nutritious food from less land and water. Meeting this challenge will require nothing short of a second Green Revolution, one that merges cutting-edge genomics with precision biotechnology. The five articles assembled in the issue “Genetic Dissection and Improvement of Crop Quality and Stress Adaptation” of Frontiers in Genetics—spanning peanut, pear, poplar, wheat, and clonally propagated polyploids—provide a timely and comprehensive blueprint for that revolution.

## From descriptive genomics to predictive breeding

Across the studies, a common theme emerges: the transition from descriptive catalogues of genes to predictive frameworks that accelerate breeding cycles. In peanut, pangenome analyses and high-throughput phenotyping platforms are being integrated to untangle the polygenic nature of drought tolerance (Pokhrel et al.). Likewise, the dissection of the EPF/EPFL gene family in poplar demonstrates how comparative genomics—anchored by *Arabidopsis* orthologues—can rapidly identify regulators of stomatal density and, by extension, water-use efficiency (Liu et al.). These efforts mirror the PAO-family characterization in pear (Ma et al.), where evolutionary analyses, cis-element profiling, and expression atlases converge to nominate candidate genes for fruit hardening and drought resilience.

Collectively, these papers underscore a critical inflection point: we are no longer constrained by the linear “gene-to-trait” paradigm. Instead, we can now leverage multidimensional data—genomic, transcriptomic, epigenomic, metabolomic, and phenomic—to build machine-learning models that predict phenotype from genotype and environment. The wheat study by Sun et al. exemplifies this synergy. By coupling morphological and ultrastructural phenotyping (root hairs, chloroplast integrity) with expression profiling of ROS-scavenging and stress-responsive genes, the authors identify diagnostic markers that distinguish salt-tolerant cultivars from sensitive ones. Such integrative approaches should become the norm, not the exception.

## Polyploid complexity as opportunity, not obstacle

Polyploidy—once viewed as a barrier to genetic dissection—is now recognized as a reservoir of adaptive potential. The review by Sakthivel et al. on vegetatively propagated crops (potato, strawberry, banana, sugarcane) reframes polyploid complexity as an opportunity for “genomic stacking” of favorable alleles. Modern genome-editing platforms, particularly CRISPR/Cas systems, can now target multiple homologous loci simultaneously, enabling trait fine-tuning without the meiotic instability inherent in traditional breeding.

Conventional introgression of late-blight resistance from wild potato species into tetraploid cultivars requires decades of backcrossing to restore tuber quality. In contrast, CRISPR-mediated multiplex editing of susceptibility genes such as *StDMR6-1* (Kieu et al., 2021) or *StNRL1* (Nourozi et al., 2023) achieves durable resistance in a single transformation cycle. Similarly, sugarcane—an aneuploid with 8–14 copies of each gene—has seen rapid progress through transgenic overexpression of *EaDREB2* or *ShGPCR1* (Ramasamy et al., 2021), as well as CRISPR editing of *SoLIM* to reduce lignin for bioethanol production (Laksana et al., 2024). These successes illustrate that high ploidy need not impede precision improvement; rather, it offers multiple genetic “entry points” for trait enhancement.

## From single-gene fixes to network engineering

Early transgenic crops relied on single-gene interventions—Bacillus thuringiensis (Bt) toxins for insect resistance or 5-enolpyruvylshikimate-3-phosphate synthase (EPSPS) for herbicide tolerance. The current wave of research, however, embraces network-level engineering. In banana, stacking RGA2 and Ced9 confers resistance to Fusarium wilt tropical race 4 (Dale et al., 2017), while simultaneous editing of endogenous banana streak virus (eBSV) loci eliminates endogenous viral sequences (Tripathi et al., 2021). In peanut, pyramiding transcription factors (DREB1A, NAC4, WRKY3) enhances drought tolerance through synergistic modulation of root architecture, osmolyte accumulation, and antioxidant capacity (Pruthvi et al., 2014; Venkatesh et al., 2022).

Such “trait stacking” must be guided by systems biology. The poplar study (Liu et al.) illustrates how EPF/EPFL peptides integrate hormonal (ABA, auxin) and environmental (drought) cues to fine-tune stomatal patterning. Future work should map these regulatory circuits across species, identifying “hub genes” whose manipulation yields pleiotropic benefits. CRISPR base editors and prime editors—capable of precise nucleotide substitutions—will be instrumental in rewiring such networks without disrupting beneficial allelic diversity.

## Democratizing technology through genotype-independent platforms

A persistent bottleneck in clonally propagated crops is genotype-dependent transformation. Sakthivel et al. highlights how transient ribonucleoprotein (RNP) delivery of CRISPR/Cas complexes can bypass this limitation, yielding transgene-free edits that are more acceptable to regulators and consumers. Such “DNA-free” editing has already been demonstrated in potato (Gonzalez et al., 2019) and strawberry (Gao et al., 2020), and its extension to banana and sugarcane will accelerate cultivar improvement in the Global South, where these crops underpin food security and livelihoods.

Equally important is the development of open-source guide-RNA libraries and multiplexed editing protocols. The wheat study (Sun et al.) exemplifies how publicly available transcriptomic datasets can be mined to prioritize candidate genes (*TaSOD1*, *TaDREB3*, and *TaWRKY19*). Global consortia—modeled after the International Wheat Genome Sequencing Consortium—should curate analogous resources for under-studied polyploids such as yam, taro, and sweet potato.

### Navigating regulatory and societal landscapes

Technical breakthroughs must be coupled with transparent regulatory frameworks. The Sakthivel et al. review delineates the divergent global policies on genome-edited crops, contrasting product-based (United States, Canada) and process-based (EU, India) approaches. As SDN-1 and SDN-2 products (transgene-free) gain regulatory exemptions, breeders can deploy edited cultivars without the protracted timelines associated with GMO approval. However, consumer acceptance remains nuanced. Surveys cited in the review reveal that labeling transparency and tangible benefits (e.g., low acrylamide potatoes, provitamin A bananas) significantly sway public opinion. Meaningful stakeholder engagement—particularly with farmers and indigenous communities—will be essential for equitable technology deployment.

## Conclusion

The convergence of genomics, phenomics, and genome editing has placed us on the cusp of a new agricultural era. The studies in this issue demonstrate that we can now dissect and re-engineer the genetic underpinnings of complex traits—drought tolerance, disease resistance, fruit quality—in species once deemed intractable. Yet technology alone will not suffice. Success will hinge on equitable access, transparent governance, and sustained investment in both basic science and on-the-ground implementation. If we rise to this challenge, the crops profiled here—peanut, pear, poplar, wheat, and their polyploid kin—will not merely survive climate change; they will thrive, nourishing a resilient and food-secure world.

## References

[B1] Dale J. James A. Paul J. Khanna H. Smith M. Peraza-Echeverria S. (2017). Transgenic Cavendish bananas with resistance to fusarium wilt tropical race 4. Nat. Commun. 8 (1), 1496. 10.1038/s41467-017-01670-6 29133817 PMC5684404

[B2] Gao Q. Luo H. Li Y. Liu Z. Kang C. (2020). Genetic modulation of rap alters fruit coloration in both wild and cultivated strawberry. Plant Biotechnol. J. 18 (7), 1550–1561. 10.1111/pbi.13317 31845477 PMC7292541

[B3] Gonzalez M. N. Massa G. A. Andersson M. Turesson H. Olsson N. Falt A. (2019). Reduced enzymatic browning in potato tubers by specific editing of a polyphenol oxidase gene via ribonucleoprotein complexes delivery of the crispr/cas9 system. Front. Plant Sci. 10, 1649. 10.3389/fpls.2019.01649 31998338 PMC6962139

[B4] Kieu N. P. Lenman M. Wang E. S. Petersen B. L. Andreasson E. (2021). Mutations introduced in susceptibility genes through crispr/cas9 genome editing confer increased late blight resistance in potatoes. Sci. Rep. 11 (1), 4487. 10.1038/s41598-021-83972-w 33627728 PMC7904907

[B5] Laksana C. Sophiphun O. Chanprame S. (2024). Lignin reduction in sugarcane by performing crispr/cas9 site-direct mutation of solim transcription factor. Plant Sci. 340, 111987. 10.1016/j.plantsci.2024.111987 38220093

[B6] Nourozi M. Nazarain-Firouzabadi F. Ismaili A. Ahmadvand R. Poormazaheri H. (2023). Crispr/Cas stnrl1 gene knockout increases resistance to late blight and susceptibility to early blight in potato. Front. Plant Sci. 14, 1278127. 10.3389/fpls.2023.1278127 38304452 PMC10830690

[B7] Pruthvi V. Narasimhan R. Nataraja K. N. (2014). Simultaneous expression of abiotic stress responsive transcription factors, atdreb2a, athb7 and atabf3 improves salinity and drought tolerance in peanut (Arachis hypogaea l.). PLoS One. 9 (12), e111152. 10.1371/journal.pone.0111152 25474740 PMC4256372

[B8] Ramasamy M. Damaj M. B. Vargas-Bautista C. Mora V. Liu J. Padilla C. S. (2021). A sugarcane g-protein-coupled receptor, shgpcr1, confers tolerance to multiple abiotic stresses. Front. Plant Sci. 12, 745891. 10.3389/fpls.2021.745891 35295863 PMC8919185

[B9] Tripathi J. N. Ntui V. O. Shah T. Tripathi L. (2021). Crispr/cas9-mediated editing of dmr6 orthologue in banana (musa spp.) confers enhanced resistance to bacterial disease. Plant Biotechnol. J. 19 (7), 1291–1293. 10.1111/pbi.13614 33934462 PMC8313124

[B10] Venkatesh B. Vennapusa A. R. Kumar N. J. Jayamma N. Reddy B. M. Johnson A. M. A. (2022). Co-expression of stress-responsive regulatory genes, munac4, muwrky3 and mumyb96 associated with resistant-traits improves drought adaptation in transgenic groundnut (Arachis hypogaea l.) plants. Front. Plant Sci. 13, 1055851. 10.3389/fpls.2022.1055851 36466254 PMC9709484

